# Short-term effects of video gaming on brain response during working memory performance

**DOI:** 10.1371/journal.pone.0223666

**Published:** 2019-10-10

**Authors:** Shuyan Liu, Christian Kaufmann, Christian Labadie, Andreas Ströhle, Maxim S. Kuschpel, Maria Garbusow, Robert Hummel, Daniel J. Schad, Michael A. Rapp, Andreas Heinz, Stephan Heinzel

**Affiliations:** 1 Department of Psychiatry and Psychotherapy, Charité –Universitätsmedizin Berlin (Campus Charité Mitte), Berlin, Germany; 2 Department of Psychology, Humboldt-Universität zu Berlin, Berlin, Germany; 3 Excellence Cluster NeuroCure, Charité –Universitätsmedizin Berlin, Berlin, Germany; 4 Department of Psychiatry (UPK), University of Basel, Basel, Switzerland; 5 Social and Preventive Medicine, Universität Potsdam, Potsdam, Germany; 6 Department of Education and Psychology, Clinical Psychology and Psychotherapy, Freie Universität Berlin, Berlin, Germany; Georgia State University, UNITED STATES

## Abstract

Breaks filled with different break activities often interrupt cognitive performance in everyday life. Previous studies have reported that both enhancing and deteriorating effects on challenging ongoing tasks such as working memory updating, depend on the type of break activity. However, neural mechanisms of these break-related alterations in working memory performance have not been studied, to date. Therefore, we conducted a brain imaging study to identify the neurobiological correlates of effects on the *n*-back working memory task related to different break activities. Before performing the *n*-back task in the magnetic resonance imaging (MRI) scanner, young adults were exposed to break activities in the MRI scanner involving (*i*) eyes-open resting, (*ii*) listening to music, and (*iii*) playing the video game “Angry Birds”. Heart rate was measured by a pulse oximeter during the experiment. We found that increased heart rate during gaming as well as decreased relaxation levels after a video gaming break was related to poorer *n*-back task performance, as compared to listening to music. On the neural level, video gaming reduced supplementary motor area activation during working memory performance. These results may indicate that video gaming during a break may affect working memory performance by interfering with arousal state and frontal cognitive control functions.

## Introduction

Breaks filled with different break activities often interweave cognitive performance in daily life. Breaks can provide respite from task-induced fatigue [1], and may enhance ongoing task performance [2, 3]. However, previous research indicates that different types of breaks, such as playing video games or listening to music, may also have deteriorating effects on ongoing tasks such as learning [4], decision-making [5], target detection [6], and working memory [7]. In particular, break activities that involve high levels of executive control and exert tension rather than relaxation seem to be more likely to affect cognitive performance negatively in the short-term [6–8]. Prior behavioural studies have identified break activities that affected executive functioning, but have not examined neural mechanisms underlying these effects [6–8]. The primary objective of this study is to investigate the neural mechanisms of effects of three typical break activities, resting, listening to music, and video gaming on executive function in a working memory updating task. Research on the effects of taking a complete wakeful rest break (restful break) from an ongoing task indicates that a restful break may improve task performance due to a positive effect on vigilance resources [6, 9]. In memory tasks, a possible performance enhancing effect of restful breaks has been attributed to the consolidation of new information [2, 3] through the reactivation of neural activities linked to recent experiences [3, 10]. Likewise, listening to music plays a critical role in preventing the mental fatigue [11] associated with improving performance in spatial reasoning and other cognitive tasks [12, 13]. More recent research, however, suggests that beneficial effects of music are rather mediated by relaxation and mood enhancement than being a specific and unique effect of classical music in itself [14]. Thalamus in the limbic system of the brain is associated with changes in emotional reactivity induced by music [11]. Research on the effects of video gaming on cognitive task performance suggests on the one hand that certain types of video gaming may enhance perceptual, attentional and cognitive skills [15, 16]. On the other hand, video games have been linked to negative effects, such as gaming-induced physiological stress [8] and impaired concentration [17] that may lead to decrements in cognitive performance.

Playing video games demand on executive control resources, which is likely to lead to a resource depleting effect of the video game on the subsequent working memory task. Thus, in line with our previous behavioural study [7], we hypothesized that video-gaming compared to restful break and listening to music would impair working memory updating (*3*-back) task performance. We expect that this effect would be modulated by subjective ratings of relaxation/tension and physiological arousal [18, 19] as previous research suggests that these factors may modulate effects of listening to music [14] and playing video games [8] on cognitive performance.

As frontal brain regions within the working memory network (mainly dorsolateral prefrontal cortex [DLPFC] and supplementary motor area [SMA]) have been frequently associated with executive control functioning (e.g. updating) within working memory [20–23], we expect reduced activations in these regions during the *3*-back performance following a short interval of video gaming compared to a restful break and listening to music.

## Materials and methods

### Subjects

Twenty-seven right-handed healthy native German subjects were recruited by advertisements in Berlin, Germany. The final sample consisted of 24 subjects (13 female; age range: 19–33, *Mean* = 24.88, *SD* = 3.57). We excluded data of three subjects from analysis: one subject with excessive interscan motion (>2 voxels translation, >1° rotation); another two subjects had performance at chance level (with either the hit rate lower than 30% or the false alarm rate more than 30%). None of the included subjects met DSM-IV criteria for any psychiatric disorder according to the screening version of the Structured Clinical Interview for DSM-IV [24]. Socio-demographic data, video gaming experience, music listening habits, and neuropsychological test results were gathered. All subjects provided written informed consent and ethics which was approved by the Ethics Committee of Charité –Universitätsmedizin Berlin (ref: EA1/132/15).

### Break activity scenarios

On three separate visits, all subjects were instructed to either engage in “eyes-open resting” (quiet rest with open eyes), “listening to music” (hearing Mozarts “Sonata for Two Pianos in D Major, KV. 448—Allegro con spirito” through headphones) or “playing a video game” (“Angry Birds” game, Rovio Entertainment, 2013) during an 8:30 min break while lying in the MRI scanner. The break was scheduled after the anatomical scan and before the fMRI scan of the *n*-back task. The break duration of 8:30 min was chosen based on the length of the musical piece and was well within the range of previous studies [2, 7]. Our choice of the Mozart Sonata KV.448 was based on previous studies investigating of the effects of music on cognitive performance [12, 25]. Angry Birds, a prominent casual game requires rapid manipulation of spatial representations combined with decisions on manual responses and has been associated with memory performance and spatial reasoning in two previous studies [26, 27].

### Procedure

**Fig 1:**
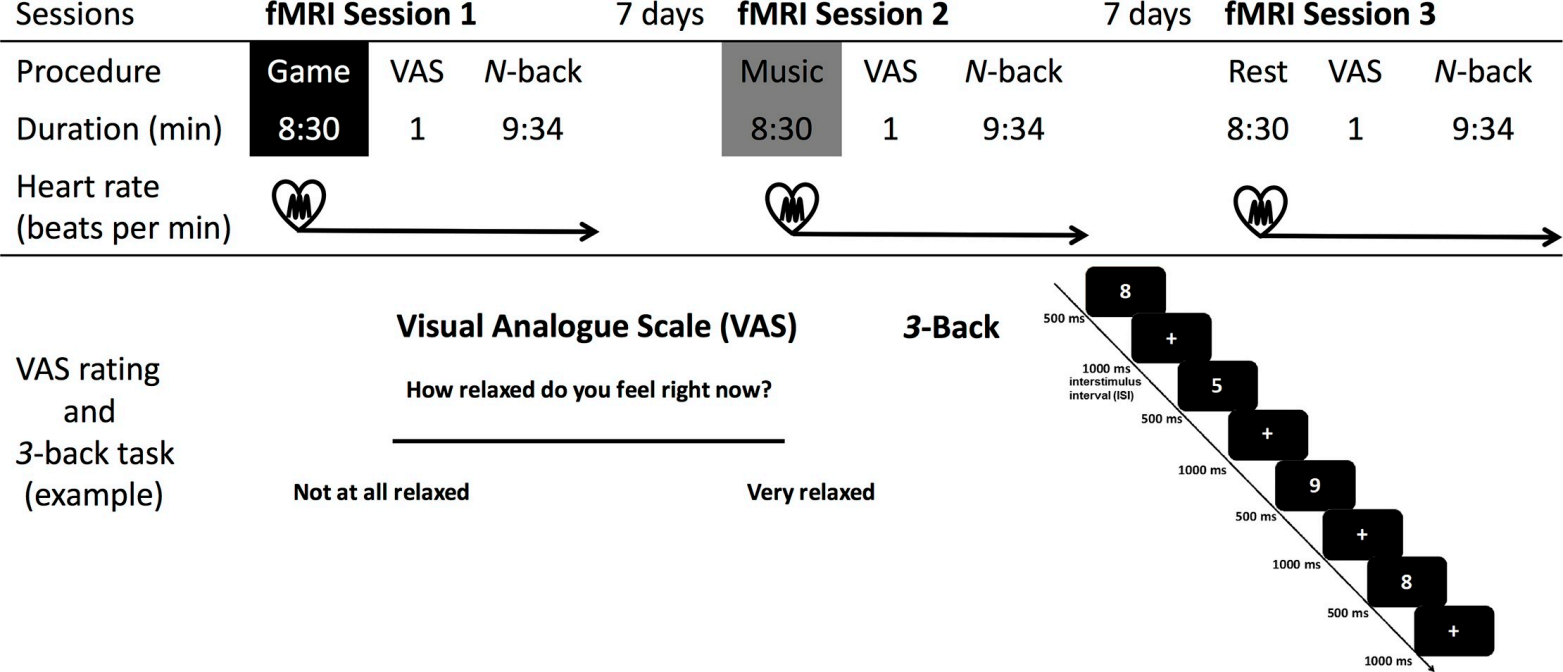
Testing procedure, visual analogue scale (VAS) rating and *N*-back task (*3*-back). Every subject underwent three fMRI scanning sessions with an intersession interval of 7 days, at consistent daytimes and weekdays. Each of the fMRI scanning sessions included: (*i*) a structural T1-weighted fMRI; (*ii*) a subsequent break of 8:30 minutes, during which subjects engaged in “eyes-open resting,” “listening to music,” or “playing a video game”; immediately after the break, subjects were asked to rate the levels of their relaxation at the moment via VAS; and finally (*iii*) the fMRI *n*-back task (using a *n*-back working memory task). In the *n*-back working memory task, random white numbers were presented on a black background for 500 ms each, followed by a white fixation cross. The length of the interstimulus interval (ISI) was 1000 ms. Heart rate data were acquired during the whole fMRI scans.

The general procedure is depicted and described in Fig 1. We utilized a modified version of an *n*-back task that we used in previous studies [28–30], presented via Presentation software® (Version 10.81, 2004, Neurobehavioral Systems Inc., Albany, CA, USA). Because ceiling effects were found in younger adults in *1*-back and, after practice, also in *2*-back, we only included *0* and *3*-back conditions [28–30]. In the *0*-back condition, subjects were required to press a response button whenever the stimulus presented was “0”. This required stimulus matching but not working memory. In the *3*-back condition, subjects had to respond if the current stimulus was identical to the stimulus presented three trials ago (see Fig 1). Stimulus duration was set to 500 ms, and the inter stimulus interval to 1000 ms.

We used a repeated-measures design with within-subjects factors for the three break activities. Subjects performed 16 blocks of *n*-back with 16 trials and five targets (31.25% of trials) within each block. Presentation order of *0*- and *3*-back was randomized across blocks and the order of break activities were counterbalanced across subjects by the use of a Latin square design. Subjects were randomly assigned to a presentation- and break order (www.randomizer.org).

Scanning consisted of three fMRI sessions with an intersession interval of 7 days, at consistent daytimes and weekdays. Each of the fMRI sessions included: (*i*) a structural T1-weighted fMRI; (*ii*) a subsequent break of 8:30 minutes, during which subjects engaged in “wakeful resting,” “listening to music,” or “gaming”; and finally (*iii*) the fMRI *n*-back task.

Before the first fMRI session, all subjects practiced *n*-back during 4 blocks with 2 blocks of each memory load (*2*- and *3*- back). Each block consisted of 20 trials (inter stimulus interval = 1500 ms). *2*-back was not used during scanning.

We asked subjects to rate the levels of their relaxation/tension on a visual analogue scale (VAS) [31] immediately after the break (i.e. “how relaxed do you feel right now?”) on a 100 mm straight line. Responses were quantified as a score indicating distance, from 0 (“not at all relaxed”) to 100 (“very relaxed”) measured in millimeters, from the “not at all relaxed” end to the subjects’ mark.

Moreover, immediately after performing the *n*-back task, subjects underwent a structured post-experiment interview. We asked subjects to rate task difficulty, their ability to concentrate on the task, as well as their motivation to perform the *n*-back task by using VAS.

### Measurement of physiological responses

To assess subjects’ physiological arousal states, we acquired heart rate data during the fMRI scans. We used the standard pulse oximeter of the physiological monitoring unit (PMU) provided by the MR scanner. The pulse oximeter scans for changes in infrared light transmission with a sampling frequency of 50 Hz. The photoplethysmograph detector was placed on subjects’ left ring finger, since subjects had to use their right hands to perform tasks. Filtering of the raw heart-rate data was performed as previously described [32], by first ignoring heartbeats that were found within the range of the pulse oximeter raw baseline (raw values below 2000) or that lead to an abrupt acceleration (more than 133%) or deceleration (less 75%) of the heartrate, most likely as a result of sudden adjustments of the hardware amplification. This filtered data were then used for determining the heartrate.

### MR image acquisition and preprocessing

We collected fMRI data at the Charité –Universitäts Medizin Berlin (Campus Mitte) with a 3 Tesla Magnetom TIM-Trio MRI scanner (Siemens, Erlangen, Germany) and a standard 12-channel head coil. Subjects were scanned while they were performing the behavioural task. At the beginning of each scanning procedure, a T1-weighted 3D "Magnetization Prepared Rapid Acquisition by Gradient Echo" (MP-RAGE) pulse sequence was obtained (repetition time (TR) = 1900 ms; inversion time (TI) = 900 ms; echo time (TE) = 2.52 ms; flip angle = 9°; field of view (FoV) = 256 × 256 mm^2^; matrix size = 256 × 256; 192 sagittal slices with 1 mm thickness; voxel size = 1 × 1 × 1 mm^3^). Functional data were obtained using a gradient echo-planar imaging (EPI) pulse sequence (TR = 2000 ms; TE = 30 ms; flip angle = 78°; matrix size = 64×64; voxel size = 3.0 × 3.0 × 3.0 mm). Using the built-in auto-align procedure of the scanner, 33 slices were acquired axial to the bicommissural plane.

### Statistical analyses of behavioural data

Statistical tests of the VAS questionnaires and heart rate data were performed using SPSS Statistics Version 24 (SPSS Inc., Chicago, IL, USA). We used a repeated-measures analysis of variance (ANOVA) with the within-subjects factors *break activity* (rest vs. music vs. game) to analyse the VAS ratings and heart rate data during breaks. When the assumption of sphericity was violated, degrees of freedom were corrected using Greenhouse-Geisser estimates. Post hoc tests were used for pairwise comparisons. To account for multiple comparisons, Bonferroni correction was used [33]. Thus, the Bonferroni-corrected threshold for significance was set to *p* < .0166 when three comparisons were performed.

To assess whether different break activities had an influence on working memory performance, we applied linear mixed-effects models as implemented in the *lme4* package [34] in the R *version 3*.*3*.*2*. system for statistical computing (www.r-project.org). The difference between hit rate and false alarm rate was used as the dependent variable “performance” for behavioural analyses. We used the predictors *memory load* (*0*- vs. *3*-back; effect coding: +0.5 vs. -0.5) and *break activity* (sliding differences contrast: game vs. rest, music vs. game; using the MASS package [35] and R-function *contr*.*sdif*) as fixed effects, as well as the interactions between these two factors. In addition, we included random subject intercepts and random subject slopes for the main effects and for the interaction, as well as random effect correlations. We tested whether the model with full random effects structure is supported by the data by comparing it to alternative baseline models: first, we set up an identical model without the random correlations; second, we also removed the random slopes; third, we fitted a model with an intercept only (in the fix and random effects). We compared the models based on log-likelihood ratios; the results were consistent when judged by the Akaike information criterion (AIC) [36] or the Bayesian Information Criterion (BIC) [37]. For statistical tests of fixed effects parameters, we used the *lmerTest* package [38].

Finally, to investigate whether the effect of break activity on task performance is modulated by subjects’ physiological arousal or relaxation level, heart rate changes during breaks and self-reported relaxation level on VAS were included separately in the best fitting model.

### MR data analyses

The brain imaging data were analysed using SPM12 (http://www.fil.ion.ucl.ac.uk/spm, Wellcome Department of Imaging Neuroscience, London, UK). First, origins in the anatomical and functional images were set manually to correspond approximately to the anterior commissure. To improve data quality, the slice repair function of the artrepair toolbox (http://www.nitrc.org/projects/art_repair/) was applied. Each subject's functional data set was head motion corrected. The T1- weighted anatomical image was then coregistered to the mean functional image per subject. After segmentation of the anatomical image, the functional data were spatially normalized into the standard MNI atlas space using the normalization parameters from the anatomical segmentation. Data were then smoothed with an 8-mm full width at half maximum Gaussian kernel and high-pass filtered during statistical analysis (128 s).

We applied the general linear model (GLM) for the MR imaging data analyses. In the GLMs, we included the experimental conditions (blocks of *0*- and *3*-back) as separate boxcar regressors of interest and other experimental conditions (the cues, responses, and six realignment parameters from the motion correction) as 8 regressors of no interest. On the single-subject level, linear contrast images were calculated for *0*- and *3*-back blocks separately for each *break activity* (rest, music and game) and contrasted against implicit baseline (fixation cross). The GLM was fitted voxelwise into the filtered time series using the restricted maximum likelihood algorithm as implemented in SPM12. On the group level, a random effects model as implemented in the GLM_Flex_Fast4 toolbox (version August 21^st^ 2015) http://mrtools.mgh.harvard.edu/index.php?title=GLM_Flex) was applied to test a repeated measures ANOVA with the within-subject factors *break activity* (rest, music and game) and *memory load* (*0*- and *3*-back). Whole brain analyses of the *break activity* by *memory load* interaction effect as well as three planned *t*-tests investigating specific effects of game vs. rest, game vs. music, and music vs. rest in brain response during *3*-back contrasted against implicit baseline were thresholded at *p* < .05, FWE cluster-level. Analyses were restricted to a working memory mask (including 26298 voxels) derived from “http://neurosynth.org” based on an automated meta-analysis of 901 working memory experiments [39]. We used a Monte Carlo simulation correction (10000 iterations) with an initial voxel-wise threshold of *p* < .001 (http://afni.nimh.nih.gov/pub/dist/doc/ program_help/3dClustSim.html; revision December 2015). We ran this analysis using a general grey matter mask that was not restricted to areas of the working memory network. Clusters with a minimum cluster size of 63 voxels that yielded a cluster-level FWE threshold of *p* < .05 are described in the results section. Behavioral and fMRI data as well as scripts for analyses are uploaded to the publically accessible open science framework homepage: https://osf.io/ed8xm/.

## Results

### Behavioural ratings

Characteristics of subjects, sociodemographic information and neuropsychological battery test results are presented in Table 1. On average, subjects spent 12.5 hours per week more on listening to music than on gaming (*t*(23) = 2.91, *p* = .008), see Table 1 for means and standard deviations). There were no frequent gamers (gaming more than 9 hours/week) according to the definition by Kühn and Gallinat [40], and no professional musicians among the 24 subjects.

**Table 1:** Sociodemographic information, characteristics of subjects, and neuropsychological test performance.

	N = 24
Age (years)	24.88 (0.73 ^a^)
Education (years)	16.73 (0.65)
Time spent on listening to music per week (hour)	13.75 (4.35)
Time spent on gaming per week (hour)	1.23 (0.28)
Fluid Intelligence Cognitive Speed (DSST) [41]	89.13 (3.20)
Verbal Knowledge (MWT-B) [42]	28.75 (0.46)
Verbal Memory (Wordlist) [43]	9.58 (0.13)
Verbal Working Memory (DS) [41]	7.79 (0.41)
Semantic Verbal Fluency (SVF) [44]	27.29 (1.36)
Executive Functioning (TMT-A, seconds) [45]	22.24 (1.06)
Executive Functioning (TMT-B, seconds) [45]	48.93 (3.01)

Note.

^a^ Standard error of the mean (SEM). Cognitive Speed was assessed by the Digit Symbol Substitution Test (DSST) from the WAIS-R; Verbal Knowledge was assessed by the German Vocabulary Test (Mehrfachwahl-Wortschatz-Intelligenztest, MWT-B); Verbal memory was assessed by Wordlist from the Consortium to Establish a Registry for Alzheimer's Disease (CERAD); Verbal Working Memory was assessed by the Digit Span (DS) Backwards Test; One-minute Semantic Verbal Fluency (SVF) tested for the category “animals” (Verbale Flüssigkeit: Tiere); Executive Functioning was assessed by the Trail Making Test (TMT-A, TMT-B).

### Behavioural ratings

During three fMRI scanning sessions with an intersession interval of 7 days each, there was no significant difference in the VAS ratings of the difficulty of three sessions of task (*F*(2, 40) = .31, *p* = .74, *η*^2^ = .02), subjects’ ability to concentrate while performing the *n*-back task (*F*(2, 40) = .028, *p* = .97, *η*^2^ = .001), and their motivation to perform the *n*-back task (*F*(2, 34) = .32, *p* = .73, *η*^2^ = .02).

**Fig 2:**
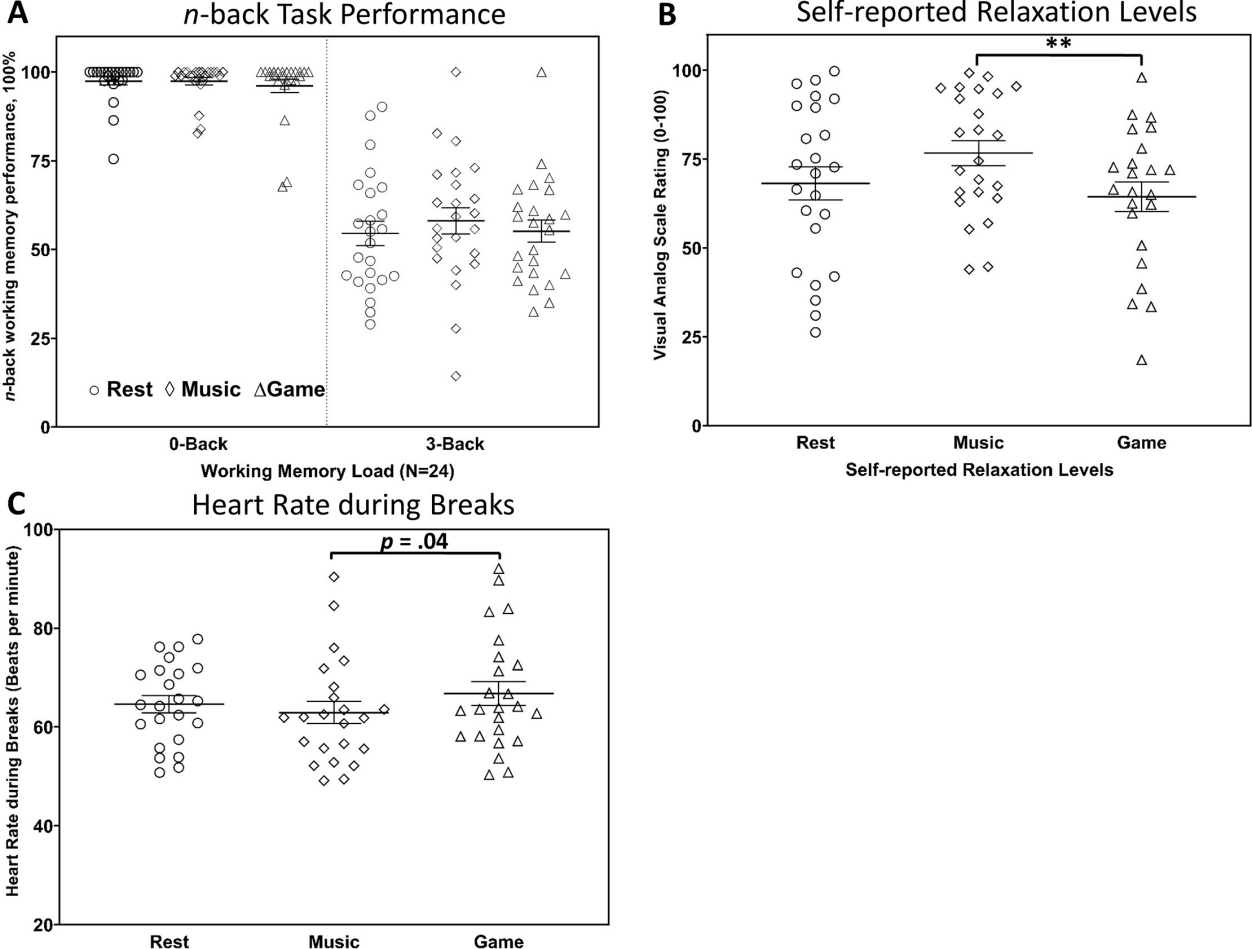
Scatterplots. **p < .0166. Please note that *p* < .0166 indicates the significance threshold after applying Bonferroni-correction for three comparisons. Horizontal lines in the scatterplots indicate means. Error bars represent standard errors of the mean. (**A**) Task performance in *n*-back as a function of memory load (*0*-back vs. *3*-back) and break activity (game vs. music vs. rest). (**B**) Self-reported relaxation levels on visual analogue scores for game, music, and rest conditions. Visual analogue scales (VAS) were adapted from Bond & Lader (1974). (**C**) Heart rate during game, music, and rest conditions.

As shown in Fig 2, the interaction of the subjective relaxation level immediately after engaging in different break activities (*F*(2, 44) = 2.89, *p* = .07, *η*^2^ = .12) did not reach significance. However, follow-up *t*-tests indicated that subjects rated themselves as 12% more relaxed (*t*(22) = 2.86, *p* = .009) after listening to music as compared to after gaming. Subjects' ratings of how relaxed they felt after eyes-open resting was not significantly different from listening to music (*t*(23) = -1.65, *p* = .11) or gaming (*t*(22) = 0.42, *p* = .68). Future studies (i.e. with higher sample sizes) are needed to further investigate the effect.

### Physiological responses

We found a significant effect of *break activit*y (*F*(2, 42) = 4.01, *p* = .03, *η*^2^ = .16) on heart rate showing that heart rate differed significantly during different breaks. Three follow-up t-tests showed, that subjects had a slightly higher heart rate while playing video games compared to listening to music (*t*(22) = 2.17, *p* = .04) and compared to rest condition (*t*(22) = 1.95, *p* = .06). Heart rate did not differ between rest and music conditions (*t*(21) = 0.77, *p* = .45).

### Working memory performance

The model with full random effect structures (see Methods section) fitted the data significantly better than alternative baseline models (for model comparisons: *p* < .001). In this best fitting model of subjects’ task performance, we neither found a significant main effect of *break activity*, nor a significant *memory load* × *break activity* interaction (all *p*s > .40), indicating that we could not detect a direct influence from breaks on task performance.

However, when including the self-reported relaxation level into the model of the behavioural data analysis, we found a significant main effect of self-reported relaxation levels on the *3*-back task performance (*β* = 4.5, *t* = 2.52, *p* = .014), as well as a two-way interaction between *break activity* and self-reported relaxation levels by comparing game either to music conditions (*β* = 9.0, *t* = 2.30, *p* = .03), or rest conditions (*β* = -5.6, *t* = -1.83, *p* = .07), showing that reduced task performance after gaming was associated with lower feelings of relaxation immediately after a break.

When including heart rate data during break activities into the model, we found that changes of heart rate during a break contributed to the interaction of break activity on *3*-back performance (*β* = 8.2, *t* = 1.97, *p* = .06), indicating that lower task performance after gaming was related to an increased physiological arousal (heart rate) during the gaming break compared to the music break, but not compared to resting (*p* > .61).

### fMRI results

**Fig 3:**
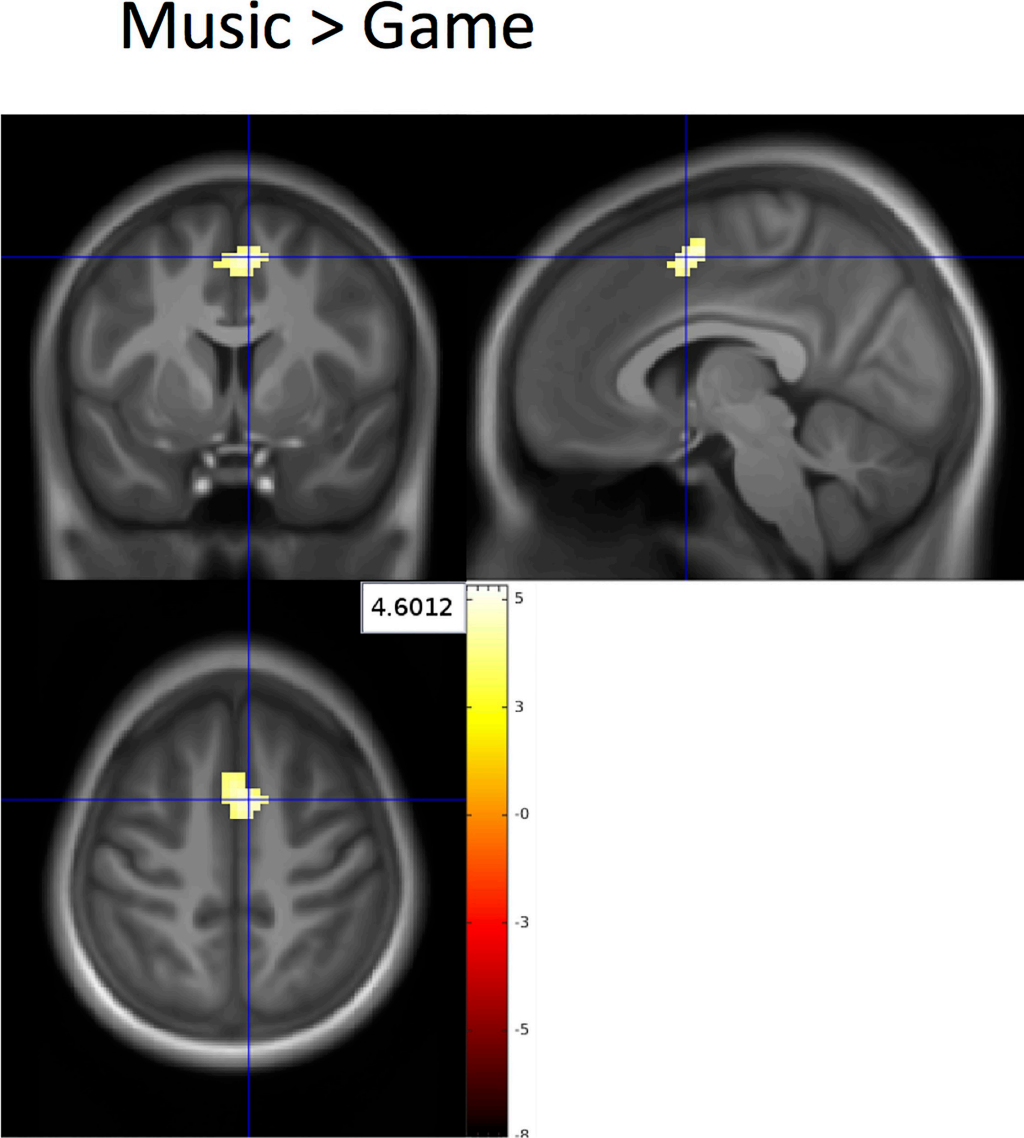
Neural correlates of break effects in the *n*-back task (*t*-tests for the *3*-back vs. implicit baseline contrast). Only results are displayed that were significant at *p* < .05, FWE cluster-corrected (*k* >62 voxel). Whole-brain results of activation changes in the bilateral supplementary motor area (SMA, peak voxel (*x* = 6, *y* = 5, *z* = 53), *k* = 67, *t*(46) = 4.60, *p* < .001) for the contrast (music > game) in *3*-back task.

BOLD response was calculated for the interaction of *break activity* (rest vs. music vs. game) by *memory load* (*0*- vs. *3*-back). Subsequently, three planned *t*-tests comparing the three break activities with each other were calculated separately for the *3*- back condition (*3*-back vs. implicit baseline contrast). All results (peak activations, cluster sizes, *t*- and *p*-values) are reported at a significance threshold of *p* < .05 FWE cluster-corrected (*k* >62 voxel) and depicted in Fig 3. No clusters surviving cluster-correction were found for the *break activity* × *memory load* interaction. However, after gaming compared to listening to music, we observed a significantly decreased activation in bilateral SMA (Fig 3). The comparisons of music vs. rest and game vs. rest did not yield cluster-correctable results.

## Discussion

When testing behavioural, physiological, and neurobiological effects of three common break activities (i.e. eyes-open resting, listening to music, and playing a video game) on working memory performance, we found that increased heart rate during gaming as well as decreased relaxation levels after a video gaming break was related to poorer *n*-back task performance, compared to listening to music. On the neural level, video gaming reduced SMA activation while performing a *3*-back task compared to listening to music.

When comparing mean levels of *n*-back performance without considering modulating parameters, we failed to detect significant differences in working memory performance between three break conditions in our sample. Possibly, our sample of young, healthy, and well-educated adults may have enough working memory resources that did not appear to be generally affected by the break activities applied in this study [46]. Future studies will have to detail performance by increasing working memory load and task difficulty and using longer periods of time preceding and following different break activities. Interestingly, however, we found inter individual differences in gaming-related increases in physiological arousal and tension that modulated decrements in working memory performance. It seems that the video game “Angry Birds” does not affect all subjects in the same way, as mainly subjects with low relaxation levels and increased physiological arousal during gaming showed reduced performance in the *3*-back task. This result is in line with a previous investigation showing that tense compared to relaxed arousal states reduced *3*-back working memory performance [18]. We found that subjects felt less relaxed and more physiologically aroused during a gaming break compared to music condition. Attentional processes may explain the “detrimental effects” of arousal on WM performance [47]. Previous reports on the “Angry Birds” game indicate that this game is specifically designed to use arousal features to motivate and engage players [26]. Conversely, Mozarts Sonata KV.448 in particular have been found to induce relaxation [48].

The main fMRI results of our study (a reduced SMA activity following gaming compared to listening to music) may be interpreted in terms of a depletion of executive control resources [49], possibly due to a strong involvement in playing the video game that may have reduced their ability to focus attention on the subsequent working memory task. Thus, playing the video game may have fatigued specific cognitive resources that rely on the SMA. In contrast to our hypothesis, no break effects on neural activity were detected in the DLPFC. It seems that video gaming may have fatigued specific SMA-related functions such as distractor susceptibility [50], and response inhibition [51], while performing the *3*-back task. A recent review [52] has indicated that these functions may be specifically vulnerable for mental fatigue effects.

Our results were limited to one particular game (Angry Birds) and one particular piece of music (Mozarts Sonata KV.448). Different types of video gaming can have different effects on cognitive functions [15, 16]. Furthermore, a relatively small sample size may limit generalizability and requires independent replication. Other measurements of physiological responses (e.g. skin conductance) could also be used to validate the contributions of arousal state.

## Conclusions

We showed that a decrease of relaxation and an increase in heart rate during video gaming led to a poorer working memory task performance compared to listening to music. Video gaming also reduced neural activations in SMA during working memory performance. Thus, specific executive control resources may be fatigued after gaming. Understanding neural underpinnings of the effects of break activities on working memory may help to guide further research into optimal resting methods, including the usage of video gaming between challenging tasks (e.g. study assignments) in young adults.
